# Flexible societies excelled in saving lives in the first phase of the COVID-19 pandemic

**DOI:** 10.3389/fpsyg.2022.924385

**Published:** 2022-08-26

**Authors:** Jianghong Li, Plamen Akaliyski, Jan Paul Heisig, Simon Löbl, Michael Minkov

**Affiliations:** ^1^President’s Research Group, WZB Berlin Social Science Center, Berlin, Germany; ^2^Telethon KIDS Institute, Perth, Western Australia; ^3^Faculty of Business and Law, Bankwest-Curtin Economics Centre, Curtin University, Perth, WA, Australia; ^4^Department of Social Sciences, Universidad Carlos III de Madrid, Madrid, Spain; ^5^Graduate School of System Design and Management, Keio University, Tokyo, Japan; ^6^Research Group “Health and Social Inequality”, WZB Berlin Social Science Center, Berlin, Germany; ^7^Institute of Sociology, Freie Universität Berlin, Berlin, Germany; ^8^Department of Business and Economics, Varna University of Management, Sofia, Bulgaria; ^9^Department of Economics, Tartu University, Tartu, Estonia

**Keywords:** COVID-19, mortality, national cultural traits, flexibility-monumentalism, mask wearing, fear of catching COVID-19

## Abstract

**Background:**

Previous studies have shown that national cultural traits, such as collectivism–individualism and tightness–looseness, are associated with COVID-19 infection and mortality rates. However, although East Asian countries have outperformed other countries in containing COVID-19 infections and lowering mortality in the first pandemic waves, no studies to date have examined flexibility-monumentalism, a cultural trait that uniquely distinguishes East Asia from the rest of the world. Moreover, none of the previous studies have explored mechanisms underpinning the association between national culture and COVID-19 mortality.

**Aims:**

Our study fills in these gaps by examining the association between flexibility-monumentalism and COVID-19 mortality, adjusting for important covariates and by analyzing mask wearing and fear of COVID-19 during the first weeks of the pandemic as plausible mechanisms underpinning this association.

**Methods:**

We constructed and analyzed a dataset including 37 countries that have valid information on flexibility-monumentalism, COVID-19 deaths as of 31 October 2020 (before the start of vaccination campaigns), and relevant covariates including two other national cultural traits (individualism–collectivism and tightness–looseness) and other national characteristics (economic, political, demographic and health). Multiple linear regression with heteroscedasticity-consistent standard errors was used to assess the independent effect of flexibility-monumentalism on COVID-19 mortality. Mediation was assessed by examining the indirect effects of flexibility through mask wearing and fear of COVID-19 and determining the statistical significance through bootstrapping. Graphical and delete-one analysis was used to assess the robustness of the results.

**Results:**

We found that flexibility was associated with a significant reduction in COVID-19 mortality as of 31 October 2020, independent of level of democracy, *per capita* GDP, urbanization, population density, supply of hospital beds, and median age of the population. This association with mortality is stronger and more robust than for two other prominent national cultural traits (individualism–collectivism and tightness–looseness). We also found tentative evidence that the effect of flexibility on COVID-19 mortality may be partially mediated through mask wearing in the first weeks of the pandemic.

## Introduction

By 22 March 2022, around 472.11 million people worldwide had been infected with COVID-19 and more than 6.09 million people had died from the disease (Ritchie et al., 2020b). However, COVID-19 mortality differs vastly across the globe: The country with the highest death rate per million was Peru, with 6,353 deaths, followed by countries in Eastern Europe, ranging from 5,268 in Bulgaria to 3,382 in Romania. Countries with the lowest death rates (<15 deaths per million population) include China, Bhutan, Burundi and Vanuatu (Ritchie et al., 2020b). These striking national variations raise a question: What accounts for them?

Research focusing on the early months of the pandemic shows that contact tracing (Kretzschmar et al., 2020), climate (Carleton et al., 2021), population aged 65 or older, the prevalence of respiratory diseases (Bosancianu et al., 2021), and income and social inequality (Bambra et al., 2020; Caul, 2020; Elgar et al., 2020; Oronce et al., 2020) were associated with country and regional level COVID-19 mortality. Single-country analyses in Germany and small-scale cross-country comparisons in Europe indicate that the timing of government interventions had an effect on the dynamics of the pandemic (Dehning et al., 2020; Brauner et al., 2021; Ram and Sornette, 2021). Despite these illuminating findings, our understanding of country differences in infection cases and mortality remains incomplete. There are substantial global variations in infection cases and mortality between countries regardless of their level of economic development or political regime type (liberal democracy vs. autocracy; Supplementary Figures S1–S4). Japan and the United States are, for example, both highly developed democracies but the pandemic took an entirely different course in the two countries. Conversely, Japan and China differ with regard to their level of economic development, political regime and government countermeasures against the pandemic (e.g., highly stringent in China and mostly non-compulsory recommendations in Japan). Yet, both countries were similarly successful in controlling the early stages of the pandemic. These observations suggest the following question: Are there some national cultural traits that explain national variations in COVID-19 infections and COVID-19 mortality rates?

Several studies have shown that national cultural traits predict COVID-19 infection and mortality rates (Salvador et al., 2020; Dheer et al., 2021; Gelfand et al., 2021; Gokmen et al., 2021; Güss and Tuason, 2021; Ozkan et al., 2021; Ruck et al., 2021): collectivism and tight social norms are associated with lower rates of COVID-19 infections and deaths, whereas cosmopolitanism is associated with higher infection and mortality rates. Scholars have also examined other prominent national cultural traits and COVID-19 infection and/or mortality rates and found significant associations between them. For example, institutional collectivism, power distance and performance orientation were associated with lower COVID-19 infection and/or mortality rates in 59 countries (Kumar, 2021), whereas higher relational mobility (a stronger community-level tendency to engage with strangers and freely choose friends) was linked to more rapid growth in COVID-19 infections and deaths in the first 30 days of the pandemic in 39 countries (Salvador et al., 2020). Schopf (2022) found that rational values (citizens’ reliance on reason for adopting novel behavioral norms rather than on authority) were very effective in containing transmission, particularly during the acceleration phases of the first two pandemic waves.

To date, no studies have examined flexibility-monumentalism (in short, flexibility; Minkov et al., 2018a), as a cultural trait that uniquely distinguishes East Asia from the rest of the world. Flexibility-monumentalism reflects cultural differences in high vs. low self-regard, self-control (self-discipline), and self-consistency (being practical and flexible in dealing with different situations vs. having an immutable self, guided by stable personal values). Compared with Western countries, East Asian countries tend to exhibit greater cultural flexibility; they also outperformed Western countries in containing COVID-19 infections and avoiding or lowering excessive mortality in the first pandemic phase.

This raises two questions: Can flexibility as a national trait explain global variations in COVID-19 mortality? If so, what might be the mechanisms through which flexibility may have played a role in shaping the COVID-19 pandemic outcomes? There is some evidence that national cultural traits are associated with antipathogenic behaviors, such as wearing masks (Lu et al., 2021) and social distancing (Chen et al., 2021). While these studies explore the connection between national cultural traits and individual behaviors that can influence the course and consequences of the pandemic, they do not examine whether and how preventative behaviors, such as wearing masks, may mediate the association between national culture and COVID-19 mortality. Thus, our knowledge remains limited in terms of understanding the *full pathway* from national culture to the severity of the pandemic.

A further limitation of previous studies of national culture and the COVID-19 pandemic is a reliance on Hofstede’s measure of collectivism–individualism, which is problematic for several reasons. This measure was based on data collected more than 50 years ago from non-representative samples of IBM employees. It misclassifies East Asian countries as highly collectivist, and it places the United States and other English-speaking countries at the top of the individualism ranking, which has not been confirmed in more recent studies (Takano and Osaka, 1999; Schwartz, 2006; Takano and Sogon, 2008; Welzel, 2013; Beugelsdijk and Welzel, 2018; Minkov et al., 2018a; Minkov and Kaasa, 2021a). Due to these limitations of Hofstede’s measure of collectivism–individualism, existing findings concerning the effect of collectivism on COVID-19 morbidity and mortality might be biased either upward or downward. Even though individualism is associated with lower prevalence of mask wearing (Lu et al., 2021), it remains puzzling that East Asian societies, ranking around the middle of the individualism–collectivism scale according to more recent data (including Japan, China, and South Korea), were exceptionally successful in dealing with the early phase of the pandemic, while more collectivist cultures in Latin America (e.g., Peru, Colombia, Mexico) failed to combat the pandemic effectively. This raises the question of whether another cultural dimension, such as flexibility-monumentalism, that captures the distinction between East Asia and Latin America, could add value in explaining global variation in COVID-19 mortality.

Our study aims to bridge these gaps in the existing research linking national culture to the COVID-19 pandemic by answering two research questions: Does flexibility-monumentalism as a newly discussed national cultural trait (Minkov et al., 2018a) predict cumulative COVID-19 mortality? If yes, do mask wearing and fear of catching the COVID-19 virus explain some of the effect of flexibility-monumentalism on COVID-19 mortality? To answer these questions, we test two hypotheses. First, we hypothesize that flexibility-monumentalism is negatively associated with COVID-19 mortality: In societies ranking higher on flexibility, mortality rates are lower than in countries ranking lower on flexibility (higher on monumentalism) (Hypothesis 1). We further hypothesize that aggregate levels of individual risk perception (fear of catching the disease) and preventive behaviors (wearing masks) account for much of the effect of flexibility on COVID-19 mortality (Hypothesis 2). Below we discuss the rationale and motivation for each of these hypotheses.

Hypothesis 1 is based on the following theoretical and empirical literature. While the cultural dimension of flexibility-monumentalism is conceptualized and measured as a continuum (rather than a dichotomy), it is best described in terms of its opposing poles. At the flexibility pole, often exemplified in East Asian societies, individuals are encouraged to be adaptable to shifting circumstances (Minkov et al., 2018a), promoting strong emotional management (Minkov et al., 2018b), adaptability, and investment in self-improvement (Hofstede, 2001; Minkov and Hofstede, 2012). In societies ranked high on the flexibility scale, one of the main goals in the socialization of children is to help them develop an ability to exercise self-control, especially an ability to suppress desires that distract from the pursuit of a superordinate goal, as well as suppression of negative feelings (Hofstede, 2001; Minkov et al., 2018b), which translates into a stronger ability to live with discomfort. There is a famous saying in China, a country ranked high on the flexibility scale: “Eat bitter first and taste sweet later.” “Eat bitter first” means that one must do hard work or even endure suffering in the present time in order to “taste sweet” (obtain rewards) in a bright future. Flexible societies promote strong emotional management, adaptability, and investment in self-improvement (Minkov et al., 2018b). At the monumentalism pole (e.g., Latin America and Africa), the human self is, figuratively speaking, like a proud and unchangeable monument. Rather than being malleable and adaptable, the individual is expected to be invariant and tethered to immutable values and beliefs (Minkov et al., 2018a,b). Parents’ preferred advice for children is to follow their natural impulses, to satisfy their desires rather than to suppress them, to give vent to their feelings rather than to control them, and “to be only yourself” rather than to strive for change by becoming like “those who know more” (Minkov et al., 2018b).

Minkov and Kaasa (2021a) show that flexibility has a very close equivalent in a dimension of “objective” culture, extracted from national statistics reflecting real behaviors. Societies with flexible cultures (e.g., East Asia) have high educational achievement, low violent crime rates, low adolescent fertility, and low paternal absenteeism (low percentages of children growing up without their fathers). Monumentalist societies have the opposite tendencies. There is a rich literature interpreting such differences in terms of life-history strategy (LHS) theory (Brumbach et al., 2009; Hackman and Hruschka, 2013; Sng et al., 2017; Minkov and Kaasa, 2021a). Minkov and Kaasa (2021b) explain that flexibility-monumentalism and LHS are mirror images of the same cultural syndrome: different prioritizations of long vs. short-term goals. Slow LHS has been defined as a long-term focus in behavioral strategies, whereas a fast LHS means a short-term focus (Csatho and Birkas, 2018). In particular, LHS has been described as the balance between devoting bioenergetic and material resources to somatic effort (devoted to the continued survival of the individual organism) vs. reproductive effort (devoted to the production of offspring) (Figueredo et al., 2005; Olderbak et al., 2014). In other words, both flexibility-monumentalism and LHS highlight a contrast between calibrating one’s behavior to the potential outcomes in the distant future by exercising self-control and self-discipline and a focus on living for the present (e.g., gratification of immediate desire) and a tendency for risk-taking behavior.

Hence, we expect that individuals from more flexible societies are more willing and prepared to comply with governmental directives against the COVID-19 pandemic than those from more monumentalist countries, as they are more likely to be concerned of the long-term consequences of the pandemic and are ready to endure short-term discomfort to avoid them. Previous research has found public support for even very tough government policies, such as quarantining all inbound airline passengers and locking down locations in regions hit by the infection, to be stronger in Asia-Pacific regions (Sachs et al., 2021) where more flexible societies are located.

Our Hypothesis 2, that fear of catching the infection and wearing masks are two mediators through which flexibility is associated with lower COVID-19 mortality, is based on the following considerations. Risk-aversion is more common in flexible societies with a slow LHS (Minkov and Kaasa, 2021a). During a health crisis such as the COVID-19 pandemic, people in more flexible societies may perceive a greater threat to their long-term future, have a higher anxiety, or perceive a higher risk of catching the disease than people in more monumentalist countries. This in turn motivates not only greater compliance with government directives to contain the spread of the virus but also voluntary preventative behaviors (e.g., hygienic practices, cough and talk etiquette in public spaces). Moreover, due to their adaptability (Minkov et al., 2018a), especially under an immediate global threat, such as the COVID-19 pandemic, more flexible societies may be better prepared to come up with stringent countermeasures for controlling the spread of the disease, and individuals are more likely to show strong support of these measures. Fear of catching the disease and wearing masks in public spaces are closely associated with other preventative behaviors which were commonly observed in East Asia during the early pandemic phase, such as stringent hygiene practices (hand washing), voluntary social distancing by keeping outdoor movement at its absolute minimum, and observing rules for talking to and eating with others in public spaces (authors’ own observations).

## Materials and methods

### Data

To test our Hypothesis 1, that flexibility is associated with lower COVID-19 mortality, we constructed a dataset including 37 countries that have valid data on flexibility-monumentalism, reliable data on COVID-19 deaths as of 31 October 2020 (Ritchie et al., 2020b), and key covariates. We chose 31 October as the end of the observation period for our analysis because the vaccination programs against COVID-19 commenced in many countries in November 2020, which likely confounds the relationship between national culture and COVID-19 mortality. Since reliable information on fear of the COVID-19 virus and mask wearing are not available for all countries, our test of Hypothesis 2 is based on a smaller sample of only 23 countries out of the 37 countries.

Two countries with complete data, Kenya and Nigeria, were excluded from our analysis because they both rank zero on the Index of Assessment of Civil Registration and Vital Statistics Systems (Mikkelsen et al., 2015; see also Supplementary material) and also rank at the bottom on the Index of effective Coverage of Health Services among 204 countries and territories (e.g., Hongkong, Taiwan, US Virgin Islands) 1990–2019 (Lozano et al., 2020). This suggests that the reporting of COVID-19 infections and deaths is likely unreliable for the two countries.

### Measures

#### Outcome variable

Our outcome variable is the natural logarithm of the COVID-19 mortality rate, defined as total cumulative deaths per one million population in a country as of 31 October 2020. Like several previous studies (e.g., Gelfand et al., 2021), we use the log transformation because it reduces skewness in the distribution of the outcome variable and renders it more symmetrical. At a substantive level, a log-linear specification (which implies constant relative rather than constant absolute effects) is more appropriate for modeling an outcome that is shaped by inherently non-linear (exponential) infection dynamics. We focus on deaths rather than infections because under-reporting of the COVID-19 cases was high and testing availability was low in the early phases of the pandemic even in high-income countries (Russell et al., 2020).

#### Main predictor and covariates

The measure of flexibilty-monumentalism was developed by Minkov and colleagues (Minkov et al., 2018a), using data from nearly 53,000 respondents selected probabilistically from all main geographic regions of 54 countries and territories. All national samples had an adequate representation of working and non-working populations (high and low skills, students, pensioners and unemployed) as well as sectors (government, finance, manufacturing, agriculture). The measure was computed based on seven items measuring self-stability, self-consistency, flexibility and adaptability (three items), self-enhancement and self-confidence (three items), and willingness to help others (one item). Each of these items is measured on a three-point scale (see Supplementary material: Note 2). The authors of the scale deliberately avoided Likert scales as these are known to be affected by national or regional response styles that can seriously compromise the cross-cultural comparability of the data: e.g., preferences for scale extremes in Latin America, Africa, and the Middle East vs. preferences for the middle of the scale in East Asia (Hui and Triandis, 1989; Johnson et al., 2005; Harzing, 2006). By asking the respondents to choose from two opposites, the problems associated with Likert scales are largely avoided.

To estimate the association between flexibility and COVID-19 mortality, independent of other prominent national cultural traits, we control for a recent and valid measure of collectivism–individualism developed by Minkov et al. (2017) and tightness–looseness developed by Gelfand et al. (2011). Both cultural indicators have been linked to lower COVID-19 infection and death rates (Gelfand et al., 2021; Gokmen et al., 2021; Ozkan et al., 2021). We are interested in the net effect of flexibility on COVID-19 mortality over and above economic development, political systems, demographic characteristics, and health care capacity. Hence, in the main analysis, we control for GDP *per capita* (World Bank, 2020a), the liberal democracy index (Lührmann et al., 2020), population density (World Bank, 2020b), median age of population (United Nations, 2019), and hospital beds per 100,000 population (Ritchie et al., 2020a). Data on mask wearing and fear are only available for 23 out of 37 countries included in the main models. Hence, in the mediation analysis we control only for GDP *per capita*, arguably the most important potential confounder, due to a smaller sample of countries.

#### Mediators

We focus on fear and mask use as two potential mediators: fear of catching COVID-19 was defined as the percentage of people surveyed in a country who say they are “very scared” or “somewhat scared” that they will catch COVID-19. This information was obtained for three observation periods: March, April, and from February to October 2020 in 29 countries (YouGov, 2020a). Mask use was defined as the percentage of people surveyed in a country who reported wearing masks when in public places (YouGov, 2020b).

We are interested in fear of catching the virus and wearing masks during the early phases of the pandemic because early caution and preventative behaviors are more likely to mediate the hypothesized negative association between flexibility and cumulative COVID-19 mortality than later ones. Cross-national variation in preventative behavior was much larger in the early phase of the pandemic than in the later phase. For example, mask wearing has been consistently high in East Asia and other countries from Asia-Pacific since the onset of the pandemic; in contrast, in Europe and North America mask wearing was low (Sachs et al., 2021). As the virus spread more widely across the globe, increasingly more countries mandated mask wearing and social distancing, thus resulting in less cross-national variations in these behaviors. Reverse causality is another related concern: high COVID-19 infection and death rates might result in higher rates of mask wearing, not only because of government mandates but also as an individual (voluntary) precaution. Early adoption of mask wearing and other preventive behaviors might have a disproportionate effect on subsequent national trajectories if they help keep infection numbers in check and prevent infection dynamics from reaching tipping points followed by accelerated growth of caseloads (e.g., due to contact tracing becoming incomplete and ineffective; see Contreras et al., 2021).

We standardized all cultural trait variables as well as the liberal democracy index to have a mean of zero and a standard deviation of one for the sample of 37 countries. We used the same standardized score for flexibility-monumentalism in the mediation analysis to ensure comparability of coefficient estimates across samples. All other variables are included in their original metric or, where noted, as the natural logarithm thereof.

### Analytical strategy

We use linear regression models with OLS estimation and heteroscedasticity-consistent (HC3) standard errors to test our hypotheses. Due to missing observations for covariates and mediator variables, our analysis was restricted to 37 out of 54 countries with available data on flexibility. This raises a concern that the relationship between flexibility and COVID-19 mortality might be under- or overestimated in the reduced relative to the full 54 country sample. Given relatively small number of observations, one might also worry that the results could be driven by a few outlier countries. We conducted sensitivity analyses (robustness checks) using both graphical inspection and delete-one influence statistics (DFBETA) to address these concerns.

Mediation through fear of COVID-19 and mask wearing (Hypothesis 2) was assessed following the classic approach of Baron and Kenny (1986). That is, we calculated the indirect (mediating) effects of flexibility operating through fear of COVID-19 and mask wearing by first regressing the mediator on flexibility and then regressing COVID-19 mortality on flexibility, with both models including log GDP *per capita* as a control. We then calculated the indirect effect by multiplying the flexibility coefficient from the first with the coefficient for the potential mediators from the second regression. We explored both individual and simultaneous mediation by including fear and mask wearing one at a time and then including both simultaneously in the model. We note that the simultaneous mediation approach assumes that fear of COVID-19 and mask wearing capture distinct and independent mediating pathways, which may be debatable. In particular, one might see the two factors as sequential nodes on one mediating pathway where flexibility first results in greater fear of COVID-19, which then leads to increased mask wearing and eventually lower deaths. This is another reason (in addition to the very small sample size) why the results of the mediation analysis should be considered explorative and with caution. Statistical significance of the indirect effects was determined using two-sided 95% and 90% bootstrap confidence intervals, obtained by applying the percentile method to 999 bootstrap replications. We used non-parametric (cases) bootstrap, that is, bootstrap samples were created by sampling with replacement from the pool of 23 countries included in the mediation analysis. All analyses were conducted using Stata Version 15 (StataCorp, 2017) and graphs were produced using R Version 4.2 (R Core Team, 2022) and the ggplot2 package (Wickham, 2016).

## Results

### Descriptive results

**Figure 1:**
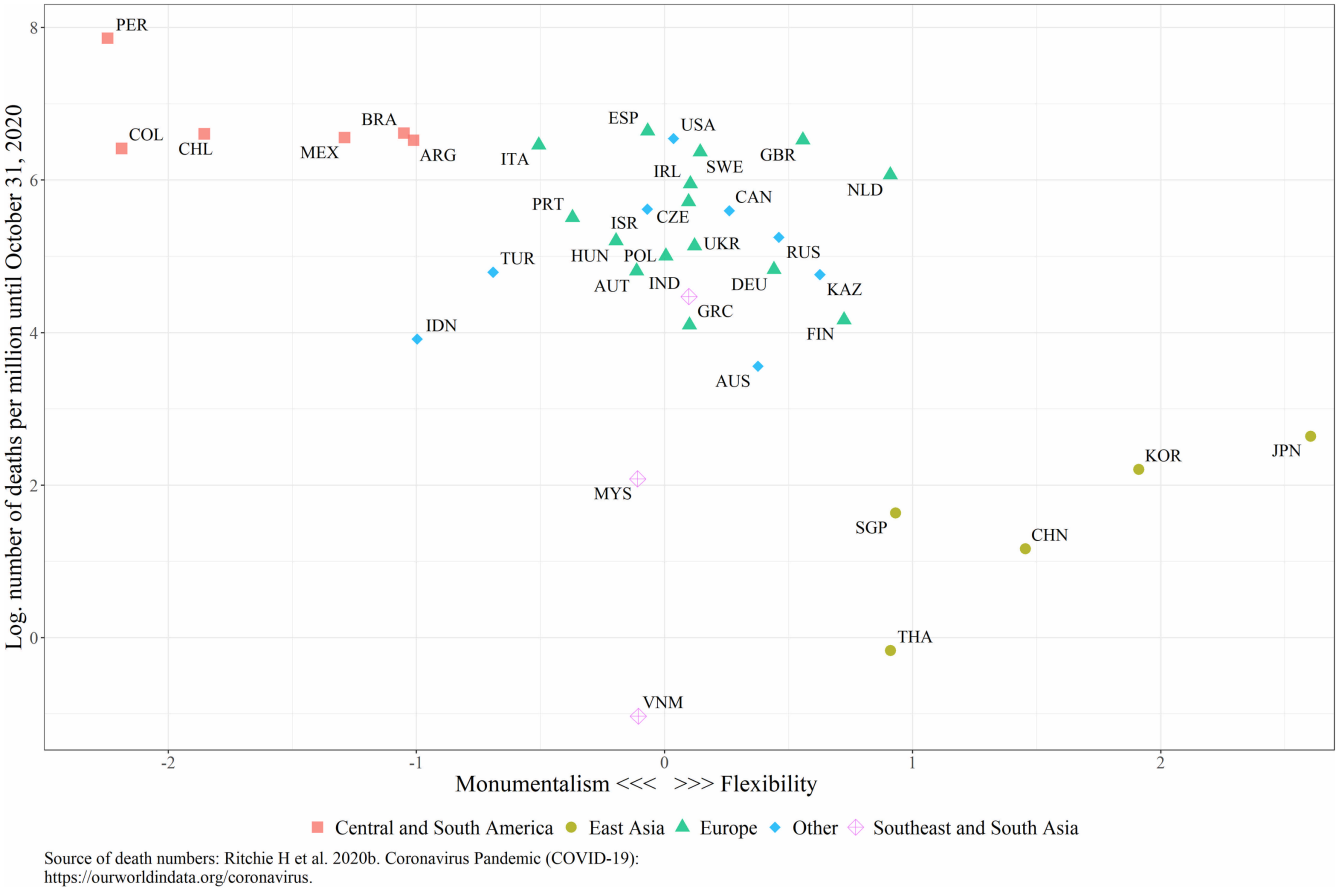
Flexibility-Monumentalism and COVID-19 mortality rate as of October 31 2020.

Figure 1 depicts log cumulative COVID-19 mortality as of 31 October 2020 for the 37 countries included in our study, with countries ranked according to their scores on the standardized scale of flexibility-monumentalism. Peru has the highest mortality rate, followed by other South American countries, the United States, and some European countries (Great Britain, Italy, Spain, Sweden). The lowest mortality is found in Vietnam, followed by Thailand, Singapore, China, Korea, Japan, and Malaysia. East Asian countries (Japan, South Korea and China) rank the highest on the flexibility scale, whereas South American countries (Peru, Columbia, Mexico, and Chile) ranked as the least flexible (most monumentalist) societies.

Table 1 shows pairwise correlations between the country-level variables in our analysis. As predicted by Hypothesis 1, the level of flexibility is significantly and negatively correlated with the cumulative mortality rate (*r* = −0.44, *p* = 0.006, *N* = 37; Table 1). Fear of catching COVID-19 and mask wearing during March 2020 are both negatively and significantly correlated with COVID-19 mortality (Table 2). While the correlations with mortality remain negative (and in the case of fear also statistically significant) when fear and mask wearing are averaged over the entire observation period from February through October in 2020, they are much weaker than for fear and mask wearing in March 2020. The bivariate correlations between flexibility and fear and mask wearing are rather weak. The only meaningful association emerges for mask wearing in March 2020 (*r* = −0.76, *p* = 0.00002; Table 2). While these results cast some doubts about the hypothesized mediation effects, zero-order correlations should be interpreted with caution as they may be confounded or suppressed by another covariate. We therefore now turn to a series of country-level regressions that control for other national cultural traits and potential confounding variables.

**Table 1:** Correlations among COIVD-19 mortality, national cultural indicators, and covariates (*N* = 37).

	(1)	(2)	(3)	(4)	(5)	(6)	(7)	(8)	(9)
(1) Log daily cumulative number of confirmed deaths (per million people)	1.00								
(2) Flexibility (vs. Monumentalism)	−0.44^**^	1.00							
(3) Individualism (vs. Collectivism)	0.30	0.37^*^	1.00						
(4) Tightness (vs. Looseness)	−0.54^***^	0.33^*^	−0.31	1.00					
(5) Liberal Democracy Index	0.47^**^	−0.00	0.67^***^	−0.26	1.00				
(6) Log GDP *per capita*	0.31	0.39^*^	0.76^***^	−0.15	0.68^***^	1.00			
(7) Log Population density	−0.16	0.32	−0.01	0.33^*^	−0.08	0.05	1.00		
(8) Hospital beds per 1,000 people	−0.10	0.58^***^	0.31	−0.09	0.07	0.19	0.08	1.00	
(9) Median age	0.03	0.54^***^	0.69^***^	−0.11	0.47^**^	0.61^***^	0.21	0.52^**^	1.00

Flexibility (vs. Monumentalism), Individualism (vs. Collectivism), Tightness (vs. Looseness) and Liberal Democracy Index are standardized. Log Population density per sq. km of land area.

* *p* < 0.05;

** *p* < 0.01;

*** *p* < 0.001.

**Table 2:** Correlations among COVID-19 mortality, flexibility, mediators, and GDP *per capita* (*N* = 23).

	(1)	(2)	(3)	(4)	(5)	(6)	(7)
(1) Log daily cumulative number of confirmed deaths (per million people)	1.00						
(2) Flexibility (vs. Monumentalism)	−0.38	1.00					
(3) Log GDP *per capita*	0.46^*^	0.32	1.00				
(4) Average fear of catching COVID-19 in March 2020	−0.58^**^	0.05	−0.63^**^	1.00			
(5) Average mask wearing prevalence in March 2020	−0.76^***^	0.23	−0.70^***^	0.84^***^	1.00		
(6) Average fear of catching COVID-19 from February–October 2020	−0.44^*^	0.01	−0.61^**^	0.93^***^	0.77^***^	1.00	
(7) Average mask wearing prevalence from February–October 2020	−0.40	0.10	−0.58^**^	0.81^***^	0.82^***^	0.78^***^	1.00

Flexibility (vs. Monumentalism) is standardized.

* *p* < 0.05;

** *p* < 0.01;

*** *p* < 0.001.

### Multiple regression results

Model 1 in Table 3 shows that flexibility is significantly and negatively associated with logged cumulative COVID-19 deaths per million population as of 31 October 2020. This association remains significant when two other national cultural traits (tightness–looseness and individualism–collectivism) are adjusted for, with the inclusion of individualism increasing (Model 2) and the inclusion of tightness decreasing (Model 3) the strength of the association between flexibility and mortality. Model 4 includes all three cultural traits, GDP *per capita*, and the democracy index as the arguably most important controls. The magnitude of the flexibility coefficient is broadly comparable to that in Models 1–3 and remains highly statistically significant, whereas the individualism and tightness coefficients are no longer significant and substantially attenuated relative to Models 2–3. Model 5 further controls for population density, hospital beds, and median age. While this model with eight predictors may be slightly over-specified given the sample of only 37 countries, it is worth noting that the flexibility coefficient remains robust and practically unchanged relative to Model 4.

**Table 3:** National cultural traits and cumulative COVID-19 mortality (in log) as of October 31 2020.

	Model 1		*VIF*	Model 2		*VIF*	Model 3		*VIF*	Model 4		*VIF*	Model 5		*VIF*
Flexibility (vs. Monumentalism)	−1.13^***^	(0.21)	1.00	−1.54^***^	(0.25)	1.16	−0.82^***^	(0.21)	1.12	−1.09^***^	(0.29)	2.00	−1.07^*^	(0.46)	3.43
Individualism (vs. Collectivism)				1.14^***^	(0.30)	1.16				0.32	(0.48)	3.39	0.43	(0.54)	3.76
Tightness (vs. Looseness)							−0.93^**^	(0.34)	1.12	−0.57	(0.38)	1.50	−0.50	(0.33)	1.78
Liberal democracy index										0.60	(0.40)	2.67	0.63	(0.46)	2.80
Log GDP *per capita*										0.11	(0.62)	3.16	0.19	(0.74)	3.43
Log Population density													−0.09	(0.22)	1.27
Hospital beds per 1,000 people													0.07	(0.12)	2.04
Median age													−0.06	(0.07)	2.87
Constant	4.76^***^	(0.29)		4.76^***^	(0.23)		4.76^***^	(0.26)		3.68	(6.22)		5.20	(8.53)	
Adjusted-*R*^2^	0.29			0.55			0.46			0.61			0.60		
*N*	37			37			37			37			37		

Standard errors in parentheses. Flexibility (vs. Monumentalism), Tightness (vs. Looseness), Individualism (vs. Collectivism) and liberal democracy index are standardized. Log of population density per sq. km of land area. *VIF*, Variance Inflation Factor. The beta coefficients are unstandardized.

* *p* < 0.05;

** *p* < 0.01;

*** *p* < 0.001.

Predicted cumulative deaths per million people illustrate the strength of the relationship between flexibility and COVID-19 mortality. We assume normally distributed errors in calculating these predictions, as this allows us to calculate predicted values for the untransformed variable as $e^{\hat{y}+{\hat{\sigma }}^{2}/2}$ , where $\hat{y}$ denotes the predicted value of the log-transformed variable and ${\hat{\sigma }}^{2}$ is an estimate of the error variance from the log-linear regression, obtained by squaring the root mean square error (Wooldridge, 2010). According to Model 4, our preferred specification because it strikes a balance between parsimony and over-controlling for potential confounders, a country with average scores on flexibility and the other predictors would have experienced a total of 261 deaths per million people by 31 October 2020. For a country with a flexibility score one standard deviation above the mean, predicted deaths decline to 87 (a three-fold reduction), while they increase to 778 (a three-fold increase) in a country with a flexibility score one standard deviation below the mean, holding the other cultural trait measures and GDP and liberal democracy constant at their respective means.

What might be some of the mechanisms that link flexibility to lower mortality? To attempt to answer this question we now turn to the mediation models. Model 1 in Table 4 shows that the association between flexibility and mortality continues to be negative and statistically significant in the reduced sample of 23 countries with valid information on fear and mask wearing. In fact, the relationship is noticeably stronger than in the full sample of 37 countries. When fear is added to the analysis, the effect size for flexibility on COVID-19 mortality decreases, although it remains significant at *p* < 0.05 level (Table 4: Model 2). When mask wearing is added to the model, the effect of flexibility declines even more strongly and is no longer significant at the conventional level of *p* < 0.05 (Table 4: Model 3).

**Table 4:** Flexibility, mediators and cumulative COVID-19 mortality (in log) as of October 31 2020.

	Model 1		*VIF*	Model 2		*VIF*	Model 3		*VIF*	Model 4		*VIF*
Flexibility (vs. Monumentalism)	−1.95^**^	(0.64)	1.11	−1.62^*^	(0.73)	1.26	−1.13	(1.02)	2.02	−1.28	(1.06)	2.21
Log GDP *per capita*	1.26^*^	(0.52)	1.11	0.63	(0.60)	2.06	0.22	(1.06)	3.73	0.29	(1.08)	3.80
Average fear of catching COVID-19 in March 2020				−0.06	(0.03)	1.86				−0.03	(0.04)	3.80
Average mask wearing prevalence in March 2020							−0.04	(0.03)	3.54	−0.03	(0.05)	7.24
Constant	−7.99	(5.35)		1.51	(7.24)		3.74	(11.42)		4.38	(11.30)	
Adjusted-*R*^2^	0.49			0.56			0.56			0.55		
*N*	23			23			23			23		

Standard errors in parentheses. Flexibility (vs. Monumentalism) is standardized. *VIF*, Variance Inflation Factor. The beta coefficients are unstandardized.

^*^p < 0.05;

** *p* < 0.01;

*** *p* < 0.001.

We formally tested for mediation using a non-parametric bootstrap with 999 replications. Confidence intervals for the indirect effects of flexibility through fear and mask wearing (Baron and Kenny, 1986) were determined using the percentile method. We first tested each potential mediator individually. In this case, while the 95% confidence intervals for the mediation effect included zero for both fear and mask wearing, the 90% confidence interval for the reduction in the flexibility coefficient attributable to wearing masks did not include zero (confidence limits: −2.15 to 0.06), providing tentative evidence for a mediation of the flexibility effect through mask wearing. In a second step, we included both mediators simultaneously. In this case, while the indirect path through mask wearing continues to be stronger than the one for fear of COVID-19, statistical uncertainty is substantially higher and the 90% confidence intervals now included zero for both mediators.

We have also tested fear of catching the virus and mask wearing as mediators measured in the later phases of the pandemic in April and over the whole period from 24 February through 31 October 2020, controlling for GDP *per capita*. Neither of these variables is significantly associated with COVID-19 mortality. While the magnitude of the flexibility-mortality association diminishes somewhat, when these longer-term averages of fear and mask wearing are included, the significance level remains unchanged (Supplementary Tables S1, S2).

### Robustness checks

Given that the sample size for our analysis is relatively small, we conducted two sensitivity tests. First, we estimated two bivariate regression models including flexibility and log of cumulative COVID-19 mortality up to 31 October 2020: one model with the 37 countries with valid information on all variables under investigation and one model with 50 countries with data only for the flexibility measure and COVID-19 mortality. Four out of the original 54 countries or territories were excluded from the sensitivity tests due to the lack of or the poor quality of mortality data, including Puerto Rico, Kenya, and Nigeria. Hong Kong was omitted because it is under the direct jurisdiction of China. This allows us to inspect the flexibility-mortality association in a larger sample including 13 additional countries that are excluded from the main analysis due to missing data on some of the covariates. A plot of the two regression lines shows that the strength of the association between flexibility and COVID-19 is similar across the two samples: the coefficients are similar (−1.13 *SE* = 0.29 for 37 countries and −0.90 *SE* = 0.26 for 50 countries) and the 95% confidence intervals based on HC3 standard errors largely overlap (Supplementary Figure S5). Similarly, we compare the flexibility-mortality association based on 23 countries with available data on mask wearing, fear and GDP (for the mediation analysis), with the same association based on 50 countries with data only on flexibility and COVID mortality (Supplementary Figure S6). Again, the two regression lines are similar, with the two 95% confidence intervals largely overlapping.

Second, we conducted a “leave-one-out” analysis to assess the possibility that the main results supporting Hypothesis 1 are driven by highly influential country cases, using DFBETA influence statistics for the coefficient estimates in Table 3 (Model 4). The DFBETA values show the change in the respective coefficient associated with inclusion of a given country case, relative to a reduced sample excluding the case, expressed in terms of the coefficient’s standard error in the reduced sample (Fox, 2019). The lollipop plots in Supplementary Figure S7 show the DFBETA statistics from smallest (most negative) to largest. Absolute DFBETA values above 0.2 are labeled and the horizontal lines indicate the conventional cutoffs of +/− 2/sqrt(*N*) and +/− 1 for high and very high influences. While quite a few countries exceed the 2/sqrt(*N*) cutoff, the distribution is quite symmetric for flexibility and most other predictors, indicating that support for our Hypothesis 1 does not hinge on the inclusion or exclusion of individual country cases. For example, the inclusion of Vietnam with low GDP *per capita* and low mortality strongly pulls the GDP coefficient in the positive direction, relative to the coefficient estimate for GDP when Vietnam is omitted from the analysis. The GDP coefficient estimate including Vietnam is just the full-sample estimate of 0.11 reported in Table 3. But, when Vietnam is omitted, this estimate switched the sign to −0.21, with the absolute difference in the point estimates (0.32) corresponding to approximately 76% of the reduced-sample standard error (see the value of the DFBETA statistic plotted in Supplementary Figure S7). While Vietnam exerts a substantial effect on the estimated GDP coefficient, its influence is offset almost completely by India, a country with low GDP and relatively high mortality, resulting in a rather symmetric overall distribution of the DFBETA statistic for the GDP coefficient. The distribution of DFBETA statistics appears most asymmetric for individualism (Panel B). This suggests that the inclusion of Vietnam may partly explain why we find less support for a mortality-increasing effect of individualism (or, equivalently, a mortality-reducing one of collectivism) than previous studies. The beta coefficient on the standardized individualism measure indeed increases from 0.32 in Table 3 (Model 4) to 0.60 and approaches statistical significance (*p =* 0.095) when Vietnam is excluded (full results available upon request). Further explanations of the analysis are provided in Supplementary material: Note 3. In a nutshell, the effect of flexibility on COVID-19 mortality is not driven by outliers.

## Discussion

### Main findings

Our analysis shows that in countries ranked high on flexibility, cumulative COVID-19 mortality was significantly lower during the initial months of the pandemic as of 31 October in 2020 than in nations that score low on flexibility. Conversely, Latin American countries, which score high on monumentalism, have the highest mortality rates. The association between flexibility and COVID-19 mortality is independent of other prominent national cultural traits that have received much attention, namely individualism-collectivism and tightness-looseness (Dheer et al., 2021; Gelfand et al., 2021; Gokmen et al., 2021; Güss and Tuason, 2021; Ozkan et al., 2021). The association persists after counting for health care capacity and key economic, political and demographic characteristics of the countries. Individualism-collectivism and tightness-looseness are less robust to the inclusion of these covariates. Thus, our study demonstrates that flexibility-monumentalism is a unique national cultural trait that explains cross-country variations in COVID-19 mortality in 2020. The study contributes new evidence to the existing literature on the relationship between national culture and COVID-19 infection and death rates (Salvador et al., 2020; Dheer et al., 2021; Gelfand et al., 2021; Gokmen et al., 2021; Güss and Tuason, 2021; Kumar, 2021; Ozkan et al., 2021; Ruck et al., 2021; Schopf, 2022).

Our mediation analysis of 23 countries provides some evidence that the association of flexibility with COVID-19 mortality might be partly mediated through a high prevalence of mask wearing during the initial phase of the pandemic. Evidence of mediation through fear of catching the disease was weaker. Even the evidence for mediation through mask wearing should be viewed with caution, however. The coefficient on mask wearing is not statistically significant, and only the 90% (but not the 95%) confidence interval for the indirect path through mask wearing does not include zero when we test for individual mediation. Statistical uncertainty becomes even higher once we include both mediators simultaneously. It should also be noted that the estimated coefficients are unlikely to reflect an entirely causal effect of mask wearing prevalence, partly because individual mask wearing is likely to be correlated with other unobserved preventive behaviors, including hygiene measures, reduction in social contacts, or ventilation practices. It is clear that more rigorous tests and larger samples will be required to draw stronger conclusions concerning mask wearing (and fear of catching COVID-19) as potential mediators of the flexibility-mortality association.

### Limitations and future directions

Our study has several limitations. First, due to a lack of reliable and valid data for the outcome variable, the main predictors, mediators, and covariates, the sample sizes are overall rather small, particularly for the models including the mediators. Thus, the findings cannot be generalized to the global level. Nonetheless, the countries included in the main models (*N* = 37 in Table 3) are diverse in terms of political systems, economic development, national culture and COVID-19 mortality. Moreover, our robustness checks show that the relationship between flexibility and COVID-19 mortality are similar between the smaller sample of 37 countries for the main analysis, the sample of 23 countries for the mediation analysis and a larger sample of 50 countries that includes additional countries with missing information on some of the covariates.

Second, due to the small sample size, the results from the mediation tests provide only tentative evidence. There likely are other (and potentially correlated) prevention measures that may also mediate the effect of flexibility on cumulative mortality, but the data for such alternative mediators are not currently available. For example, in Japan there were many rules for fighting against the pandemic: “always wear a mask,” “avoid physical contact with others,” “do not talk loudly,” “always wash and disinfect hands.” University students were asked to strictly observe additional rules, including “cough etiquette” and “rules for eating and drinking outside the home, such as curtailing eating time, avoiding eating with more than four persons or with strangers, or eating in cramped and confined places (Keio University Student Website, 2021).

In South Korea, there was the phenomenon of “excessive preventative behaviors” driven by a high level of fear and anxiety during a health crisis like COVID-19 (Hahn, 2020). Based on Hahn’s observation, South Koreans are very flexible and strove to do everything that they could (perhaps more than “necessary”) to protect themselves from COVID-19, during a time when it was not yet clear what the right or effective measures were. Wearing masks was only one of the many voluntary prevention measures taken, wearing gloves and transparent outfits when going outside being some other examples. It is not surprising that in the absence of a lockdown, South Korea brought the transmission dynamics under control during the high tide of the pandemic in March 2020. Future research should strive to consider other prevention measures than mask wearing, if such data become available.

Finally, we have only examined the relationship between flexibility and COVID-19 mortality cross-sectionally. Longitudinal analyses could further elucidate how cultural traits dynamically shape national trajectories over multiple waves characterized by emerging virus mutations and increasing availability of vaccines, and whether cultural orientations themselves might change as a result of the pandemic.

## Conclusion

Our analysis shows that flexibility-monumentalism is a unique national cultural trait that helps explain cross-country variations in COVID-19 mortality during the first phase of the global pandemic. It contributes new evidence to the emerging literature on the relationship between national culture and COVID-19 infection and death rates. It is well-known that East Asian and Pacific countries have outperformed the rest of the world when it comes to saving human lives during the first year of the COVID-19 crisis. Our analysis indicates that flexibility may provide a fundamental cultural explanation for why and how East Asians were so successful in managing the crisis.

Our study bears both research and practical implications. From the point of view of research, further investigations of the pathways from having a national cultural trait of flexibility-monumentalism to saving human lives during a health crisis are critically needed. In order to deepen the conceptualization of why and how national culture influences the devastating outcomes of a global crisis like the COVID-19 pandemic, we need a better understanding of a multitude of plausible mechanisms that underpin this influence. This will require a sound conceptualization and valid operationalization of these mechanisms as both single and composite measures in future research. We hope our study can stimulate this further inquiry.

In terms of practical implications, we still face a great deal of uncertainty about the nature of COVID-19 (Karim and Karim, 2021; Rubin et al., 2021). We are into the third year of the COVID-19 pandemic, and yet we witness new and more contagious variants of the virus continuing to emerge and spread globally, oftentimes outpacing vaccination programs and dampening our hope to return to normality sooner rather than later. Current vaccines do not provide bullet-proof protection against COVID-19. The vaccine-induced immunity wanes over a short time, particularly in high-risk groups such as the elderly. Thus far vaccination has been unable to prevent “breakthrough” infections, permitting subsequent transmission to other people, even though it reduces severe and fatal disease (Morens et al., 2022). Experts warn that it remains unknown whether and how permanent immunity can be achieved, and whether it can prevent emergence of immunity-escaping variants of COVID-19 (Morens et al., 2022).

Thus, before new vaccines with broader protective efficacy and more durable immunity are developed, non-pharmaceutical public health measures such as mask wearing, personal hygiene and reducing physical contact will continue to play an important role in curbing the transmission of the disease (Karim and Karim, 2021). National cultural traits and norms are likely to continue to influence the progression and the consequences of the COVID-19 in the foreseeable future. The lessons we learn from this pandemic may also help increase our preparedness for future pandemics.

## Data availability statement

The dataset and the codes used for all of the analyses are available through this link: https://github.com/loeblsim/FlexCov.

## Ethics statement

Ethical review and approval was not required for the study on human participants in accordance with the local legislation and institutional requirements. Written informed consent for participation was not required for this study in accordance with the national legislation and the institutional requirements.

## Author contributions

JL played a leading role in the conceptualization, including hypothesis development, analysis design and modeling strategy, and writing the original draft of the manuscript. PA contributed to the conceptualization of the study and to the analysis design, and writing of the background and hypothesis. JPH has contributed to the formal analysis with regards to formal sensitivity analysis for the robustness of the results, and writing of the methods and results section. SL complied the data from various sources for the study and mainly responsible for the formal data analysis and graphic presentations of the results. MM has contributed to the formulation of the hypotheses linking the national cultural trait, flexibility vs. monumentalism, to COVID mortality, and reviewed and edited earlier drafts of the manuscript. All authors contributed to the article and approved the submitted version.

## Conflict of interest

The authors declare that the research was conducted in the absence of any commercial or financial relationships that could be construed as a potential conflict of interest.

## Publisher’s note

All claims expressed in this article are solely those of the authors and do not necessarily represent those of their affiliated organizations, or those of the publisher, the editors and the reviewers. Any product that may be evaluated in this article, or claim that may be made by its manufacturer, is not guaranteed or endorsed by the publisher.
