# Supplementary material for: Flexible societies excelled in saving lives in the first phase of the COVID-19 pandemic
Source: Front Psychol. 2022 Aug 26;13:924385. doi: 10.3389/fpsyg.2022.924385 (PMC9461707; doi:10.3389/fpsyg.2022.924385)

Supplementary Material

Notes

1. The Index of Assessment of Civil Registration and Vital Statistics Systems

This index was based on the summary index of Vital Statistics Performance (the VSPI metric) developed by Phillips et al. (2014), using the Global Burden of Disease (GDB) 2013 Study database, monitoring 148 countries between 1980 and 2012. The measure comprises six different dimensions of data accuracy (completeness of death reporting, quality of death reporting, level of cause-specific detail, internal consistency, quality of age and sex reporting and data availability). It uses simulation to determine each dimension and its weighted impact on the summary index of vital statistics performance. The VSPI proves to be a reliable metric for assessing vital statistic systems in several empirical studies (Mikkelsen et al., 2015; Phillips et al., 2015). Note on the VSPI metric".

Phillips, D. E., Lozano, R., Naghavi, M., Atkinson, C., Gonzalez-Medina, D., Mikkelsen, L., et al. (2014). A composite metric for assessing data on mortality and causes of death: the vital statistics performance index. *Popul Health Metrics* 12, 14. doi: 10.1186/1478-7954-12-14.

Phillips, D. E., AbouZahr, C., Lopez, A. D., Mikkelsen, L., de Savigny, D., Lozano, R., et al. (2015). Are well functioning civil registration and vital statistics systems associated with better health outcomes? *The Lancet* 386, 1386–1394. doi: 10.1016/S0140-6736(15)60172-6.

Mikkelsen, L., Phillips, D. E., AbouZahr, C., Setel, P. W., de Savigny, D., Lozano, R., et al. (2015). A global assessment of civil registration and vital statistics systems: monitoring data quality and progress. *The Lancet* 386, 1395–1406. doi: 10.1016/S0140-6736(15)60171-4.

2. Items measuring flexibility vs monumentalism (higher scores entail stronger flexibility)

1. I have some good qualities or skills that most other people do not have. 2. I am somewhere here, in between these two. 3. I am an ordinary person, like most other people.
1. I like to compete with people. 2. I am somewhere here, in between these two. 3. I hate to compete with people
1. Most of the good things that happen to me come from my own actions. 2. I am somewhere here, in between these two. 3. When something good happens to me, I feel it is just good luck.
1. I would feel bad if I had to pretend and act like a different person. 2. I am somewhere here, in between these two. 3. I can pretend that I am somebody else without feeling bad about that.
1. I am usually the same person at home and outside (at work, at school, in public places). 2. I am somewhere here, in between these two. 3. I am often quite different at home and outside.
1. I have strong values and beliefs. They guide my behavior in most situations. 2. I am somewhere here, in between these two. 3. My behavior depends on the situation, not so much on my values and beliefs.
1. I like to help people, even if I have to do something difficult. 2. I am somewhere here, in between these two. 3. I rarely agree to do something difficult to help people.

Flexibility-monumentalism was also studied in a related project of socialization goals (Minkov, Dutt, et al., 2018) where respondents were presented with a list of different types of advice that they are likely to give to their children. Parents in flexible societies preferred to advise children to suppress

their desires and their negative feelings, to feel ashamed in case of failure, and to try to be like those who know more. Parents in monumentalist societies preferred the opposite types of advice: children should try to satisfy their desires, express all feelings, downplay failure, and "be only themselves". Evidently, flexible societies promote strong emotional management, adaptability, and investment in self-improvement, whereas in monumentalist societies the norm is to be true to one's nature and avoid attempts to suppress it or change it, not even if the ultimate goal were self-improvement. Of note, the two studies - of flexibility vs monumentalism and of preferred advice to children - yielded very similar national scores (Minkov, Dutt, et al., 2018).

The samples of nations for operationalizing the flexibility vs monumentalism scale were nationally representative in every respect except education. In the developing countries, about two thirds of the respondents had higher education. However, separate analyses across respondents without higher education, with higher education and mixed show minimal differences in the dimension structures and country rankings. Furthermore, the samples had an adequate representation of all professions in a country (high and low skills, students, pensioners and unemployed) and sectors (government, finance, manufacturing, agriculture, etc.).

3. The “leave-one-out” analysis.

We conducted a “leave-one-out” analysis to assess the possibility that the main regression results are driven by highly influential country cases, focusing on DFBETA influence statistics for the coefficient estimates in Table 1, Model 4. The DFBETA values, depicted in Figure 6, can be seen as the change in the respective coefficient associated with inclusion of a given country case (relative to the reduced sample omitting the country case), expressed as multiples of the associated standard error in the reduced sample. For example, the DFBETA value of -.42 for Thailand in panel A means that the full-sample estimate for the flexibility is “more negative” in the full sample than in the reduced sample omitting Thailand, and the magnitude of this differences is equal to 42% of the flexibility coefficient’s standard error in the reduced sample.

Commonly used cutoffs for considering a data point are absolute DFBETA statistics greater than 2/sqrt(*N*) – approximately .33 given our sample size of 37 – or greater than one, with the former criterion being the more conservative one. It is also important, however, not to mechanically apply these cutoffs on a case by case basis but to also consider the overall distribution of the DFBETA. A situation where the latter is symmetric such that influential cases drawing the coefficient estimate in one direction are counterbalanced by influential cases drawing it in the other is less worrisome than one where the distribution is strongly asymmetric.

The plots in Figure 1 show the DFBETA statistics from smallest (most negative) to largest. Absolute DFBETA values above .2 are labeled and the horizontal lines indicate the cutoffs of +/- 2/sqrt(*N*) and +/- 1. None of the DFBETA statistics exceed the +/-1 threshold. While quite a few do break the 2/sqrt(*N*) cutoff, the distribution is quite symmetric for most predictors. For example, Vietnam (a country with low GDP per capita and low mortality) strongly pulls the GSP coefficient in the positive direction, but this is offset by the case of India (a country with low GDP and relatively high mortality). More specifically, the GDP coefficient changes from .110 (full-sample estimate) to -.215 when Vietnam and to .562 when India is excluded, but is very similar to the full-sample estimate when both countries are dropped simultaneously (.115). The one case where we find rather asymmetric distribution of DFBETAs is the individualism coefficient, which is drawn in the negative direction by Vietnam with no other country case having a similarly large impact in the opposite

direction. The individualism, indeed almost doubles from .319 (full-sample estimate) to .602 when Vietnam is excluded. However, it does not quite attain statistical significance when in the latter case (se = .349, p (two-sided) = .095).

Figure S1. Liberal democracy and COVID-19 mortality rates.

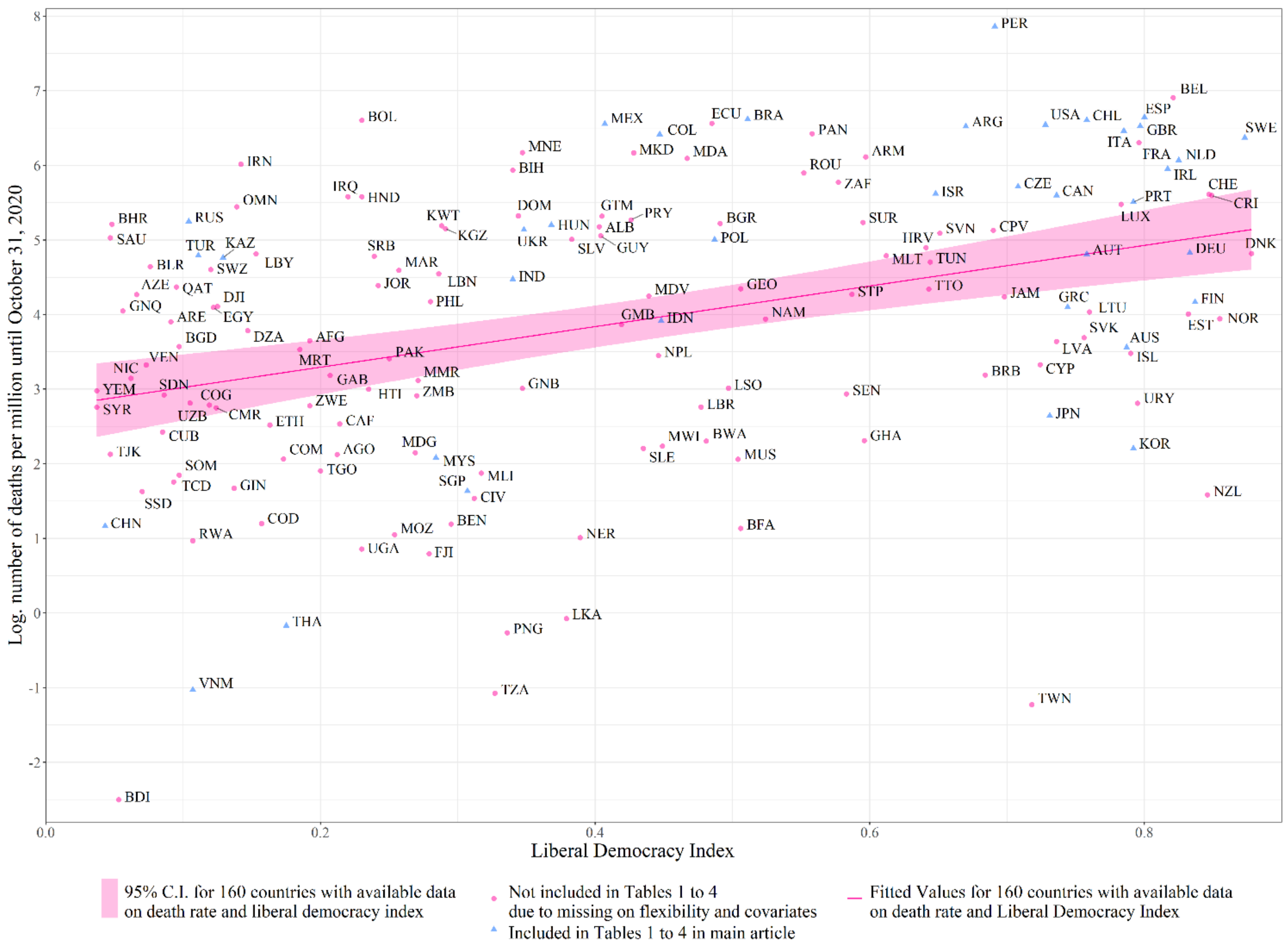

Source of death numbers: Ritchie H et al. 2020b. Coronavirus Pandemic (COVID-19): https://ourworldindata.org/coronavirus.

Figure S2. Liberal democracy and COVID-19 infection rates.

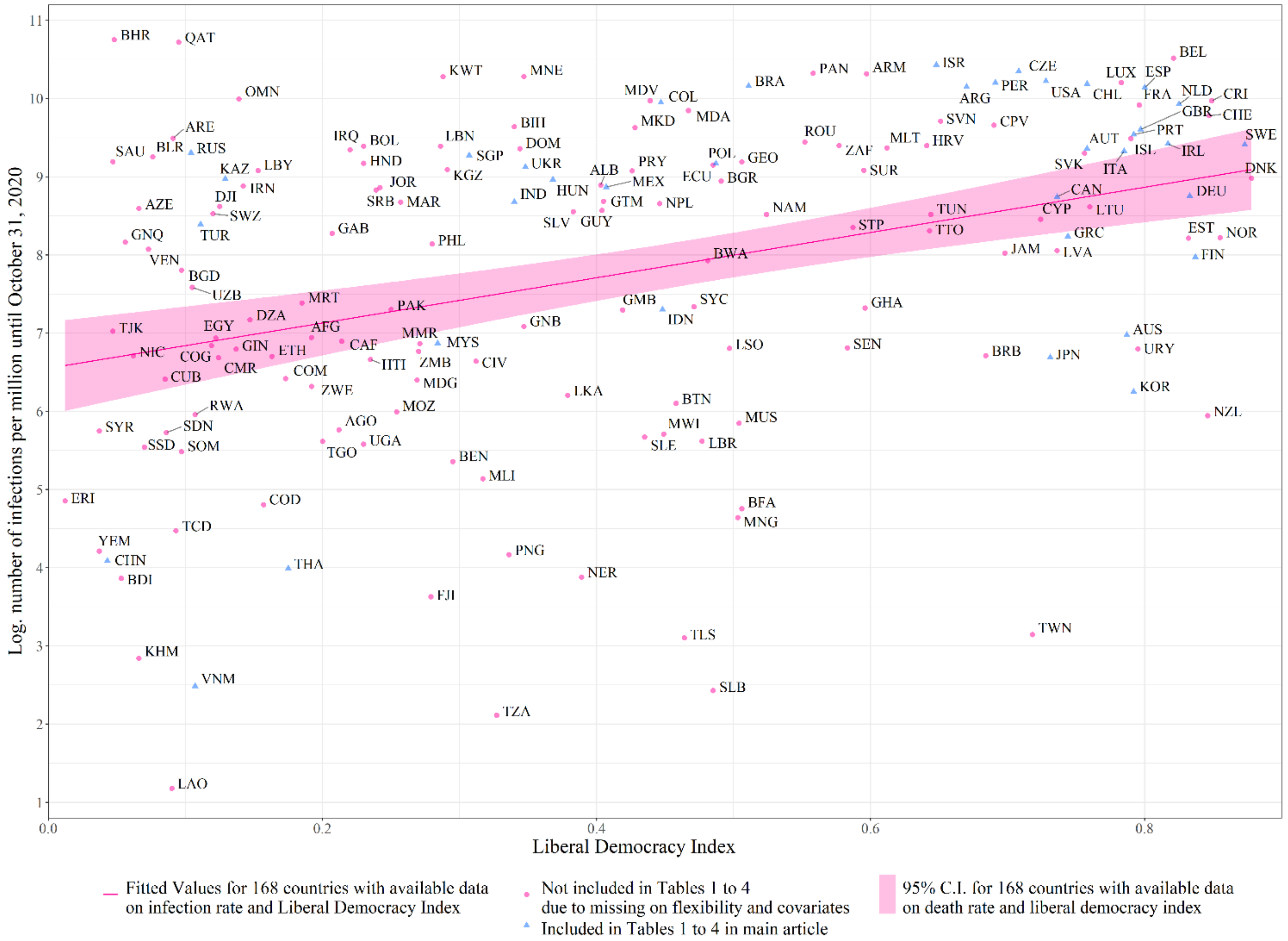

Source of infection numbers: Ritchie H et al. 2020b. Coronavirus Pandemic (COVID-19): https://ourworldindata.org/coronavirus.

Figure S3. GDP per capita and COVID-19 mortality rates.

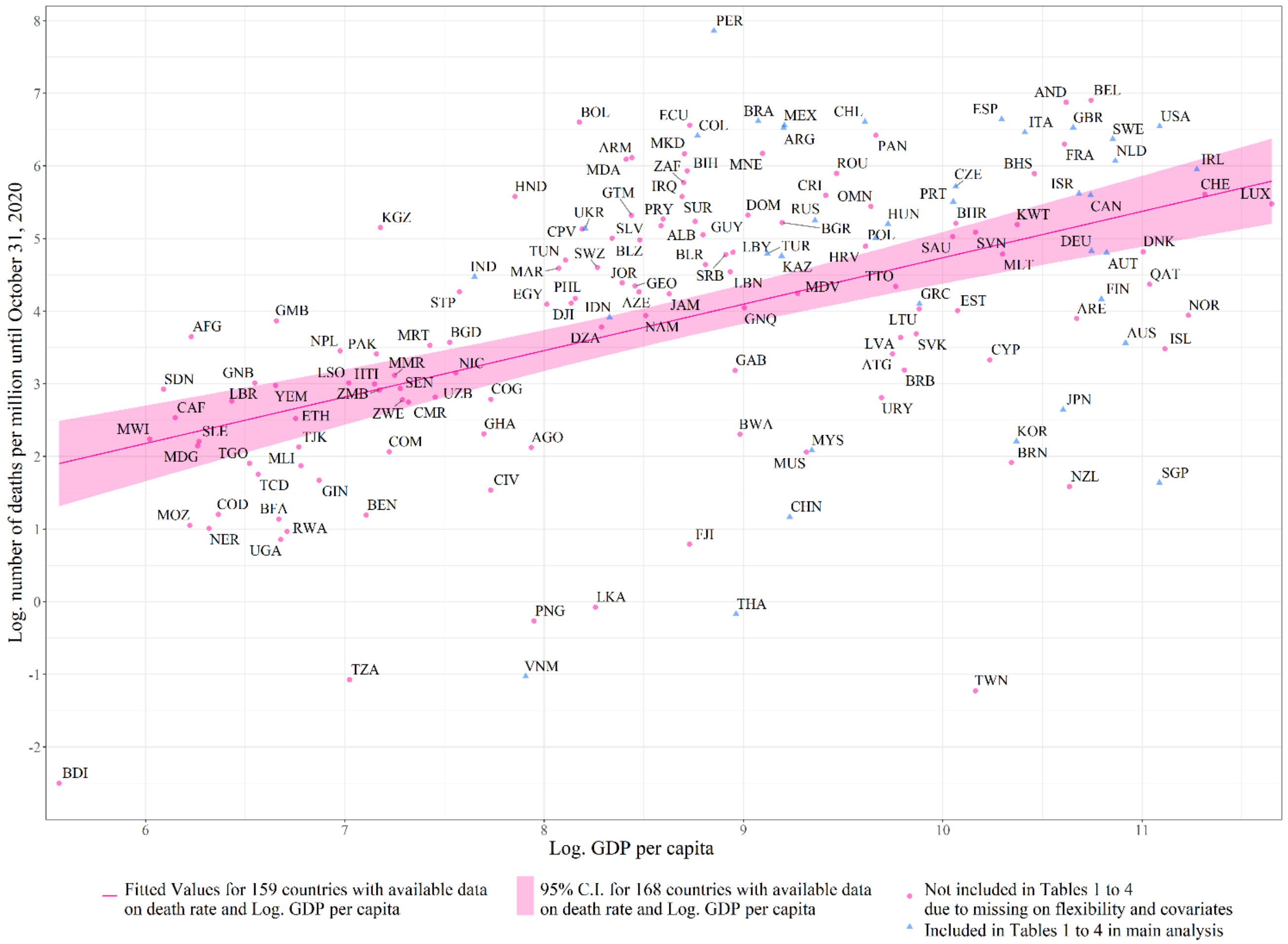

Source of death numbers: Ritchie H et al. 2020b. Coronavirus Pandemic (COVID-19): https://ourworldindata.org/coronavirus.

Figure S4. GDP per capita and COVID-19 infection rates.

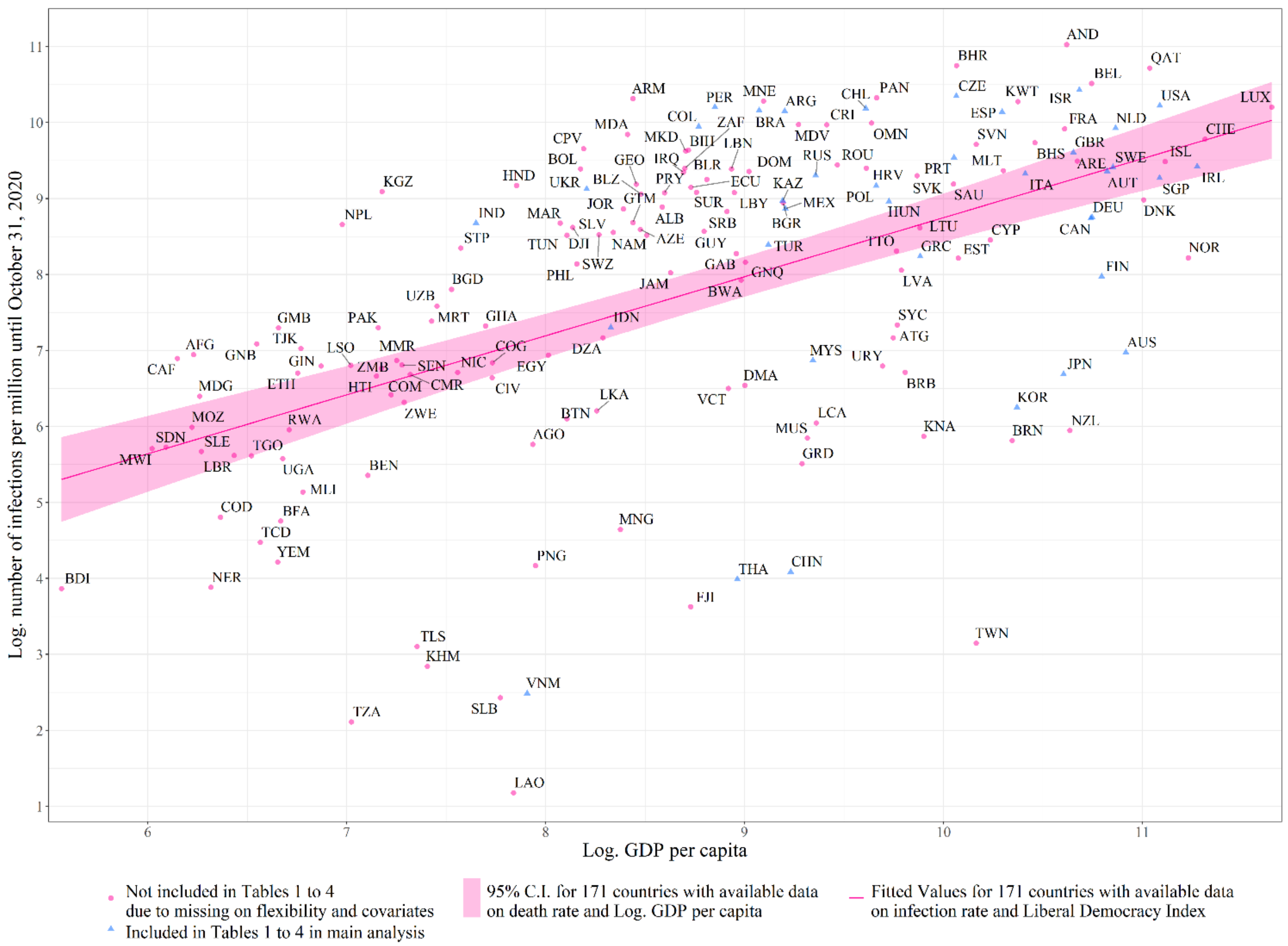

Source of infection numbers: Ritchie H et al. 2020b. Coronavirus Pandemic (COVID-19): https://ourworldindata.org/coronavirus.

Table S1. Flexibility, mediators and cumulative COVID-19 deaths (in log) as of October 31 2020.

	Model 1		*VIF*	Model 2		*VIF*	Model 3		*VIF*
Flexibility (vs. Monumentalism)	-1.95**	(0.64)	1.11	-1.81*	(0.79)	1.20	-1.90**	(0.64)	1.29
Log GDP per capita	1.26*	(0.52)	1.11	0.93	(0.70)	1.92	1.18	(0.68)	1.93
Average fear of catching COVID-19 from February-October 2020				-0.04	(0.04)	1.73			
Average mask wearing prevalence from February-October 2020							-0.00	(0.02)	1.75
Constant	-7.99	(5.35)		-2.64	(8.99)		-6.93	(7.54)	
Adjusted-R^2	0.49			0.49			0.46		
N	23			23			23		

Note: Standard errors in parentheses. Flexibility (vs. Monumentalism) is standardized. *VIF* = Variance Inflation Factor. * $p < 0.05$, ** $p <.01$, *** $p <0.001$.

Table S2. Flexibility, mediators and cumulative COVID-19 deaths (in log) as of October 31 2020.

	Model 1		*VIF*	Model 2		*VIF*	Model 3		*VIF*
Flexibility (vs. Monumentalism)	-1.95**	(0.64)	1.11	-1.67*	(0.74)	1.37	-1.73*	(0.68)	1.33
Log GDP per capita	1.26*	(0.52)	1.11	0.90	(0.64)	1.88	0.87	(0.75)	2.33
Average fear of catching COVID-19 in April				-0.04	(0.04)	1.74			
Average mask wearing prevalence in April							-0.02	(0.02)	2.11
Constant	-7.99	(5.35)		-2.00	(8.19)		-3.19	(8.27)	
Adjusted-R^2	0,49			0.50			0,49		
N	23			23			23		

Note: Standard errors in parentheses. Flexibility (vs. Monumentalism) is standardized. *VIF* = Variance Inflation Factor. * $p < 0.05$, ** $p <0.01$, *** $p <0.001$.

Figure S5. Robustness check for main models.

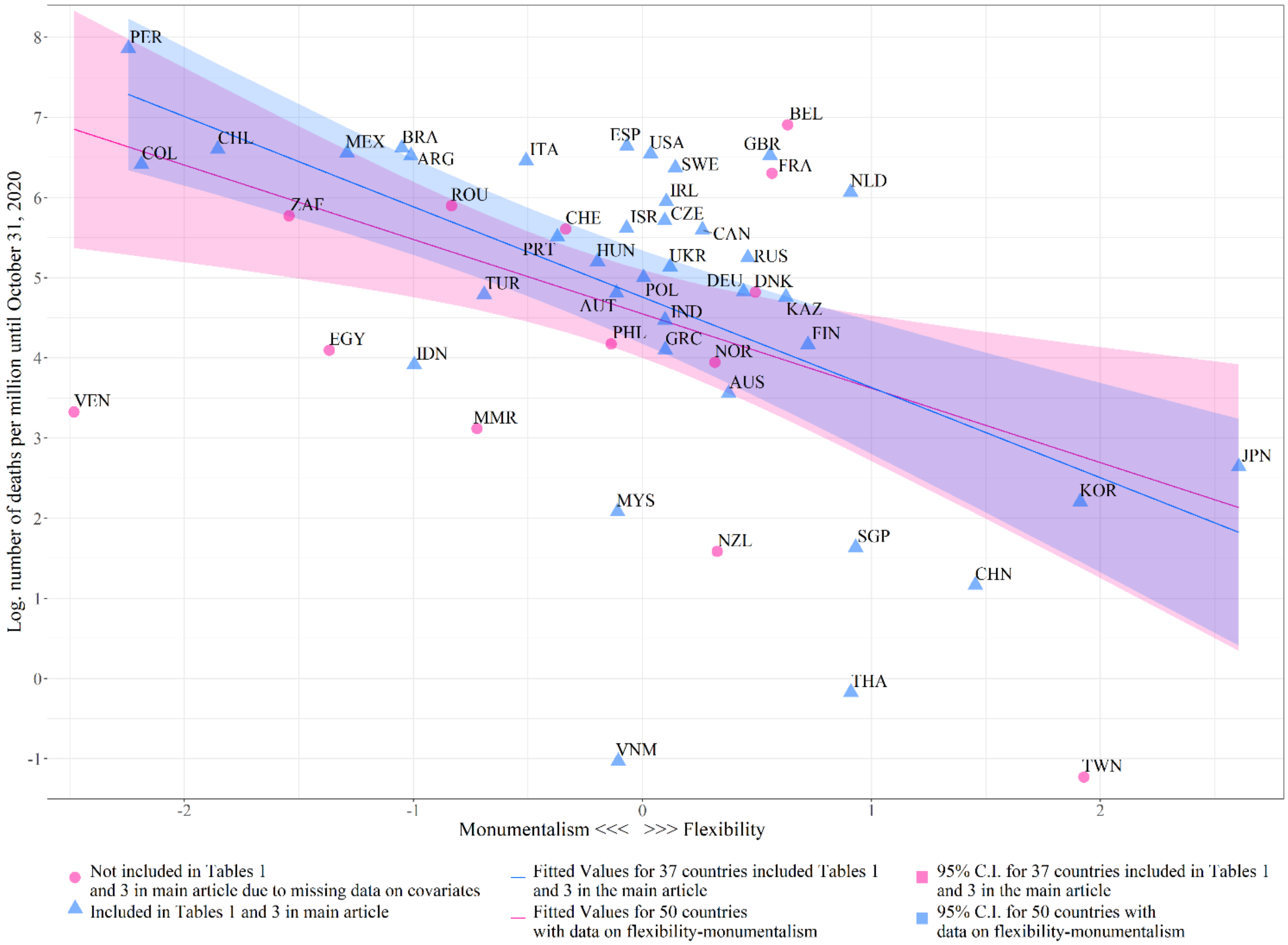

Source of death numbers: Ritchie H et al. 2020b. Coronavirus Pandemic (COVID-19): https://ourworldindata.org/coronavirus.

Figure S6. Robustness check for mediation models.

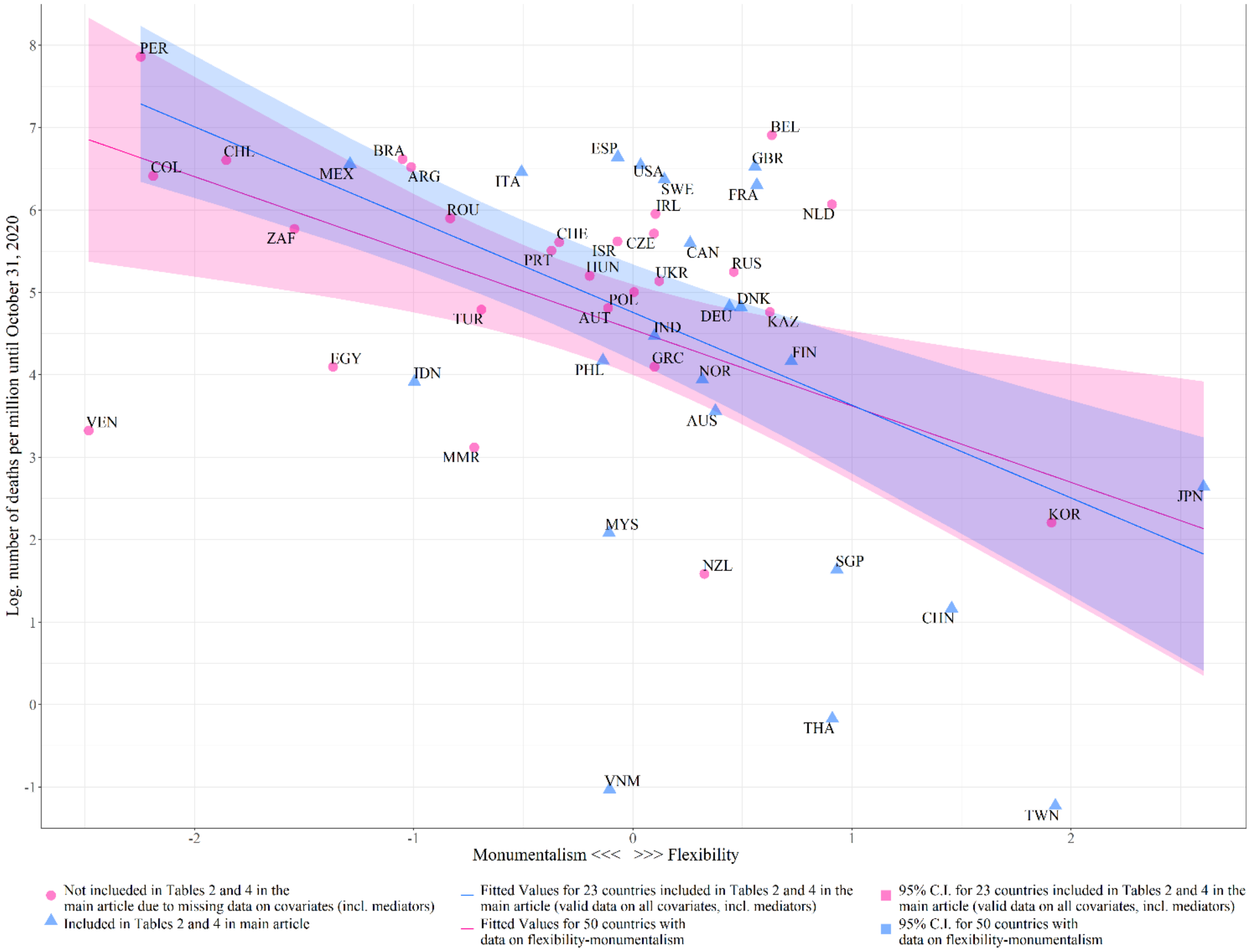

Source of death numbers: Ritchie H et al. 2020b. Coronavirus Pandemic (COVID-19): https://ourworldindata.org/coronavirus.

Figure S7. Robustness check for outliers.

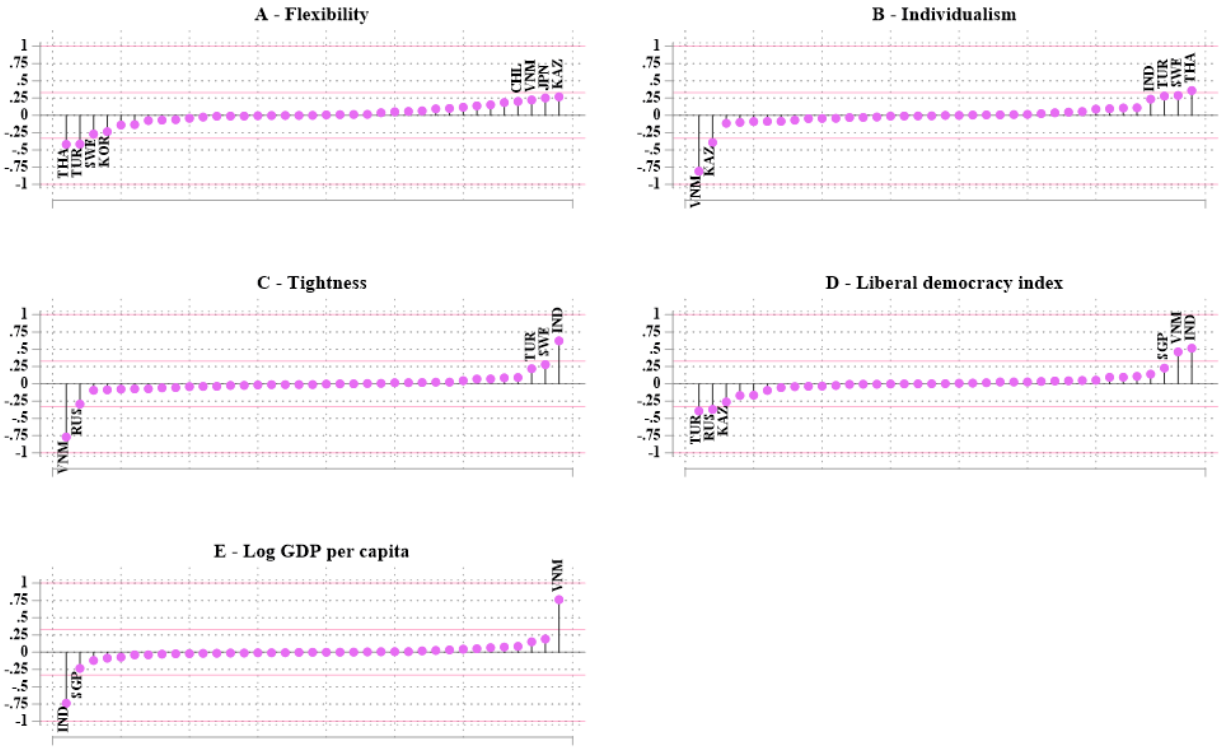

Supplement: Supplementary file 1 [file Data_Sheet_1.pdf]
